# Effects of different intrusion patterns during anterior teeth retraction using clear aligners in extraction cases: an iterative finite element analysis

**DOI:** 10.3389/fbioe.2024.1388876

**Published:** 2024-06-06

**Authors:** Yiyan Zhang, Kaixin Wang, Mengyu Li, Cuiyu Liu, Li Tang, Chunyan Wan, Cunhui Fan, Yang Liu

**Affiliations:** ^1^ Department of Orthodontics, The Affiliated Hospital of Qingdao University, Qingdao, Shandong, China; ^2^ School of Stomatology, Qingdao University, Qingdao, Shandong, China; ^3^ Department of Endodontics, The Affiliated Hospital of Qingdao University, Qingdao, Shandong, China

**Keywords:** clear aligner appliance, orthodontic space closure, finite element analysis, biomechanical phenomena, tooth movement

## Abstract

**Background:**

Overtreatment design of clear aligner treatment (CAT) in extraction cases is currently primarily based on the clinical experience of orthodontists and is not supported by robust evidence on the underlying biomechanics. This study aimed to investigate the biomechanical effects of overtreatment strategies involving different maxillary anterior teeth intrusion patterns during anterior teeth retraction by CAT in extraction cases.

**Materials and methods:**

A finite element model of the maxillary dentition with the first premolar extracted was constructed. A loading method of clear aligners (CAs) based on the initial state field was proposed. The iterative method was used to simulate the long-term orthodontic tooth movement under the mechanical load exerted by the CAs. Three groups of CAs were utilized for anterior teeth retraction (G0: control group; G1: incisors intrusion group; G2: anterior teeth intrusion group). Tooth displacement and occlusal plane rotation tendency were analyzed.

**Results:**

In G0, CAT caused lingual tipping and extrusion of the incisors, distal tipping and extrusion of the canines, mesial tipping, and intrusion of the posterior teeth. In G1, the incisors showed minimal extrusion, whereas the canines showed increased extrusion and distal tipping tendency. G2 showed the smallest degree of posterior occlusal plane angle rotation, while the inclination tendency of the canines and second premolars decreased.

**Conclusion:**

1. In CAT, tooth displacement tendency may change with increased wear time. 2. During anterior teeth retraction, the incisor intrusion pattern can provide effective vertical control for the lateral incisors but has little effect on the central incisors. Anterior teeth intrusion patterns can alleviate the inclination of canines and second premolars, resulting in partial relief of the roller-coaster effect.

## 1 Introduction

The increasing preference for clear aligner treatment (CAT) among orthodontists and patients is attributed to its superior esthetics, comfort, and convenience (Gu et al., 2017). Owing to their complex biomechanics, clear aligners (CAs) have become a research hotspot in the field of orthodontics. CAs were initially used in mild to moderate malocclusion cases with high predictability of relieving mild crowding, closing scattered spaces, and achieving molar distalization (Djeu et al., 2005; Simon et al., 2014; Lanteri et al., 2018). CAs are increasingly being used in more complex cases, such as presurgical orthodontic and extraction cases (Dai et al., 2021; Cong et al., 2022). A recent clinical study found that the efficacy of CAT in achieving different types of tooth movement was inconsistent (Haouili et al., 2020), and unexpected movements may occur (Simon et al., 2014). Fiori et al. showed a high predictability of transversal arch expansion using clear aligners; however, a discrepancy between the virtual arch forms obtained at the end of digital planning and those obtained at the end of the aligner sequences has been reported (Fiori et al., 2022). Evidence indicates that there are differences between predicted tooth movement and achieved tooth movement, and that an overtreatment design can optimize the biomechanical effects of CAs (Dai et al., 2019).

Premolar extraction is commonly used to treat dental protrusion malocclusion (Alkadhi et al., 2019; Alqahtani et al., 2020). However, tooth extraction treatment involves tooth movement over a long distance and the roller-coaster effect often results during the process of tooth extraction space closure, primarily manifesting as lingual inclination and extrusion of incisors, distal inclination and extrusion of canines, and mesial inclination and intrusion of the second premolars. Therefore, the predictability of tooth movement in tooth extraction cases is limited (Dai et al., 2021).

To alleviate the adverse effects of the roller-coaster effect and improve treatment efficiency, a reverse-curved stainless steel archwire is typically used in fixed orthodontic treatment. Compared with the fixed appliance, the CA has weaker three-dimensional control over tooth movement during the closure of the extraction gap and the roller-coaster effect is more obvious (Chen et al., 2022). The same phenomenon has also been recently observed in cases of CAT with premolar extraction; in these cases, overtreatment has been recommended to alleviate the roller-coaster effect (Dai et al., 2019; Ren et al., 2022). In CAT, orthodontists usually adopt overtreatment patterns, such as anterior teeth intrusion and crown-labial torque, to reduce the roller-coaster effect when performing anterior teeth retraction in extraction cases (Peretta and Ghislanzoni, 2015; Zhu et al., 2019). However, the overtreatment design of CAT in extraction cases is primarily based on the clinical experience of orthodontists and lacks sufficient biomechanical evidence. To date, no high-quality clinical studies have evaluated the effectiveness of different overtreatment patterns in CAT. In general, the biomechanisms of overtreatment in CAT during the bodily retraction of anterior teeth requires further study and discussion.

Finite element analysis (FEA) is a validated mechanical computational simulation technique that has previously been used in complex biomechanical analysis and is widely applied in orthodontic biomechanical research (Liu et al., 2022a). A previous study showed that initial displacement can predict long-term orthodontic tooth movement (OTM), which is currently the most widely used index in FEA to evaluate the biomechanics of CAs (Soenen et al., 1999). However, Hamanaka et al. noted that tooth movement can result in a change in the positional relationship between the center of resistance and the force line, such that initial displacement cannot accurately evaluate OTM (Hamanaka et al., 2017). Recently, the iterative method has been used to simulate the OTM directly in CAT (Lyu et al., 2023; Mao et al., 2023). In this study, a finite element model of anterior teeth retraction with CAs was constructed, and a novel loading method based on the initial state field was proposed. This study used the iterative method to investigate the biomechanical effects of various overtreatment strategies involving different anterior teeth intrusion patterns during anterior tooth retraction.

## 2 Materials and methods

### 2.1 Finite element model construction

This study was approved by the Medical Ethics Committee of the Affiliated Hospital of Qingdao University (approval number: QYFY WZLL 28198). Cone beam computed tomography (CBCT) data from an adult volunteer in the Department of Orthodontics of the Affiliated Hospital of Qingdao University were analyzed. A man with natural teeth; intact dentition; and no evident abnormalities in tooth size and shape, apparent dental defects, and periodontal disease was selected.

The CBCT data of the patient were extracted utilizing Mimics software (Materialise, Leuven, Belgium). Geometric models of the teeth, periodontal ligament (PDL), attachments, and CAs were established and assembled using the reverse engineering software Geomagic Wrap 2019 (Raindrop Geomagic, NC, United States) and CAD software Solidworks (Dassault Systems, United States). The thickness of the PDL was set at 0.25 mm (Liu et al., 2022b), while the CA had a thickness of 0.5 mm (Liu et al., 2021). The attachment specifications have been previously described (Liu et al., 2022a). Subsequently, the assembled geometric model was imported into the CAE preprocessing software Hypermesh (Altair, Troy, Mich, United States) and meshed with first-order tetrahedral elements (C3D4) with a size of 0.15 mm (Kwak et al., 2023). Finally, the FE model was imported into the software Abaqus/CAE 2019 (SIMULIA, Providence, RI) for analysis.

The geometric nonlinear setting was activated, assuming that all the materials were homogeneous, isotropic, and linearly elastic (Liu et al., 2023b). The material properties were derived from previous studies (Liu et al., 2022a; Cheng et al., 2022b), and detailed data are listed in Table 1.

**TABLE 1:** Properties of the materials used in this study.

	Young’s modulus, MPa	Poisson’s ratio
Tooth	19600	0.3
Periodontal ligament	0.67	0.45
Attachment	12500	0.36
Aligner	816	0.36

Regarding boundary conditions and contact relationships, in the initial step, the outer surface of the PDL was designated as a fixed constraint, maintaining its position and shape to accurately simulate the contact relationship between the PDL and alveolar bone. Tie constraints were established between the PDLs and teeth, and between the teeth and attachments. The contact relationships between the other surfaces are defined as hard contacts with frictionless (Barone et al., 2014; Cai et al., 2015; Cheng et al., 2022b).

**FIGURE 1:**
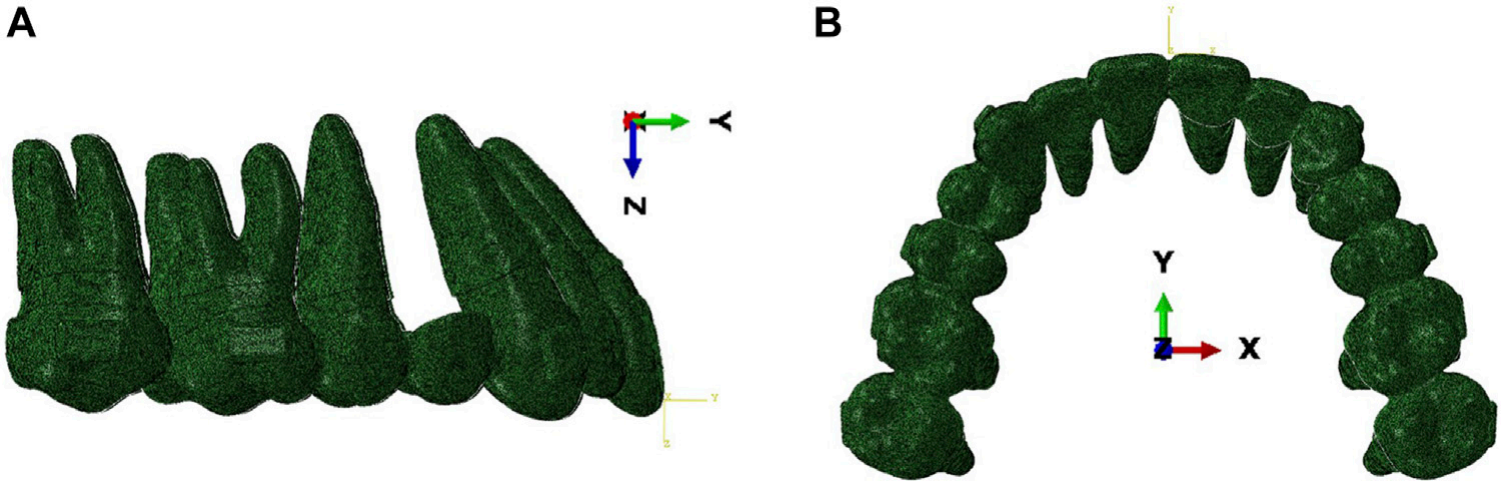
Schematic diagram of the finite element model and the global coordinate system. **(A)** Lateral view. **(B)** Occlusal view.

Additionally, a global coordinate system was established for convenient observation and analysis. Within this coordinate system, the occlusal plane was represented by the XY plane, in which the Y-axis corresponded to the symmetrical axis of the dentition, denoting the sagittal direction, with the buccal direction considered positive. The X-axis represented the leftward direction as positive, whereas the Z-axis, which is perpendicular to the XY plane, indicated that the vertical direction towards the crown was positive. (Figure 1).

### 2.2 Working conditions and loading method

The working conditions were classified into three groups (Table 2).

**TABLE 2:** Classification of working conditions.

	Designed displacement in Y-axis	Designed displacement in Z-axis
G0	−0.2 mm of anterior teeth	none
G1	−0.2 mm of anterior teeth	−0.15 mm of central and lateral incisors
G2	−0.2 mm of anterior teeth	−0.15 mm of anterior teeth

In the Y-axis direction, a positive value means labial movement and a negative value means lingual retraction; In the Z-axis direction, a positive value means extrusion and a negative value means intrusion.

In G0, or the control group, the anterior teeth exhibited a retraction of 0.2 mm along the negative direction of the Y-axis.

In G1, or the incisors intrusion group, the anterior teeth exhibited a retraction of 0.2 mm along the negative direction of the Y-axis, and the incisors experienced an intrusion of 0.15 mm along the negative direction of the Z-axis.

In G2, or the anterior teeth intrusion group, the anterior teeth exhibited a retraction of 0.2 mm along the negative direction of the Y-axis and an intrusion of 0.15 mm along the negative direction of the Z-axis.

**FIGURE 2:**
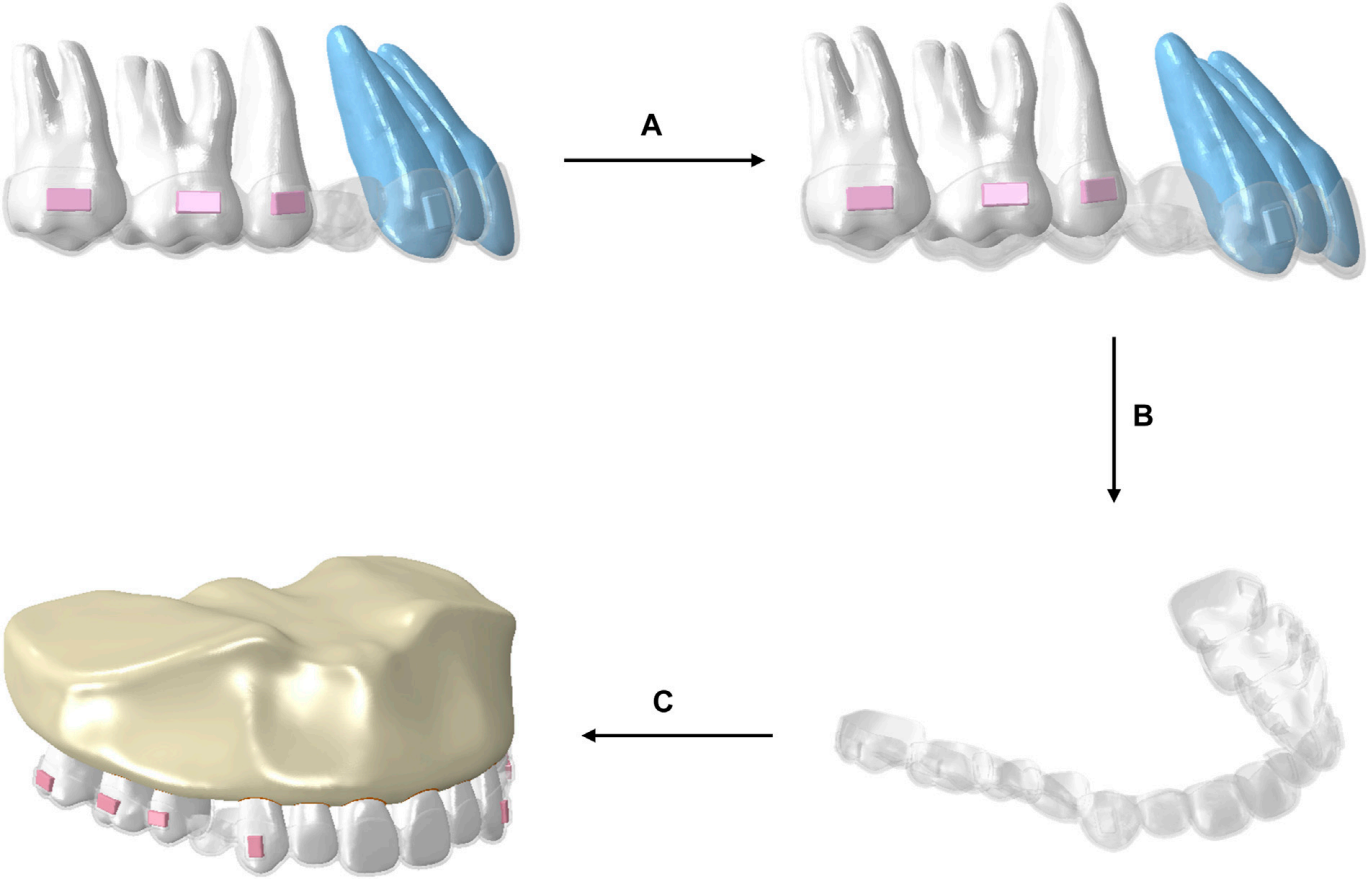
Schematic illustration of the procedural steps of the loading method of CA. **(A)** The teeth in the target locations were repositioned to their initial positions, resulting in deformation of the aligner. **(B)** The deformed aligner was obtained. **(C)** The deformed aligner was loaded onto the original dentition.

The loading process of the working conditions was divided into two sequential steps (Figure 2). The first step involved reversing the teeth from target positions to initial positions, and a deformed aligner was obtained. At this stage, there was no interference between the CA and initial dentition. The deformed aligner was subsequently applied to the initial dentition utilizing the initial state field.

**FIGURE 3:**
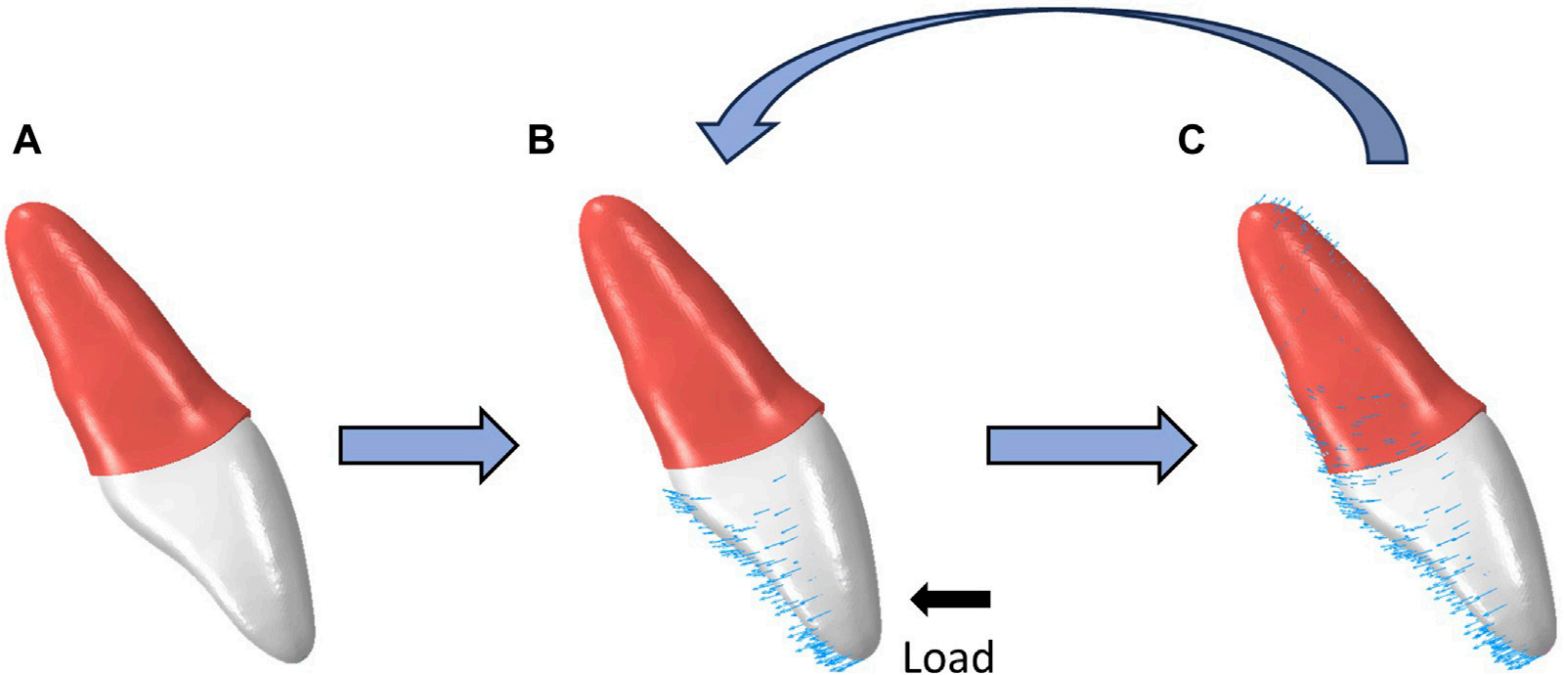
Schematic illustration of the procedural steps of OTM simulation by iterative method. **(A)** The original state of the teeth and PDLs. **(B)** The teeth subjected to mechanical loads from CAs, and the PDLs undergo deformation. **(C)** The external surfaces of the PDLs undergoes a displacement identical to that of the tooth root and subsequently resumes its initial configuration.

In this study, an iterative method was used to simulate tooth displacement after long-term application of an aligner (Yokoi et al., 2019). Initial tooth displacement was observed with the loads applied by the CAs, causing deformation of the PDLs. Then, the shapes of the PDLs were restored to their original state by applying displacement to the outer surface of the PDLs, which matched the displacements of the roots. This process was used to simulate the remodeling of the alveolar bone. The cumulative tooth displacements were calculated through repeated iterations of the above two steps (Figure 3). Based on a previous study (Lyu et al., 2023), the number of iterations was set at 15.

### 2.3 Data extraction

The central points on the incisal edges of the incisors, the cusps of the canines, and the occlusal surfaces of the posterior teeth were selected as the reference markers for tooth displacement. Coordinate changes and displacement values in the Y-axis and Z-axis of the central and lateral incisor, canines, and second premolar crowns were recorded. The following parameters were analyzed in this study: 1) the vertical and sagittal displacements with iterations were assessed to evaluate individual tooth displacement. 2) The relative vertical displacement of individual teeth at the end of iteration, calculated from the vertical crown displacement of an individual tooth minus the vertical crown displacement of the second premolar, was used to assess the vertical control of individual teeth. 3) The rotation angles of the anterior occlusal plane (AOP) and posterior occlusal plane (POP) at the end of iteration were determined by calculating the initial and deformation coordinates of the central incisor, second premolar, and second molar to evaluate changes in the occlusal plan.

## 3 Results

**FIGURE 4:**
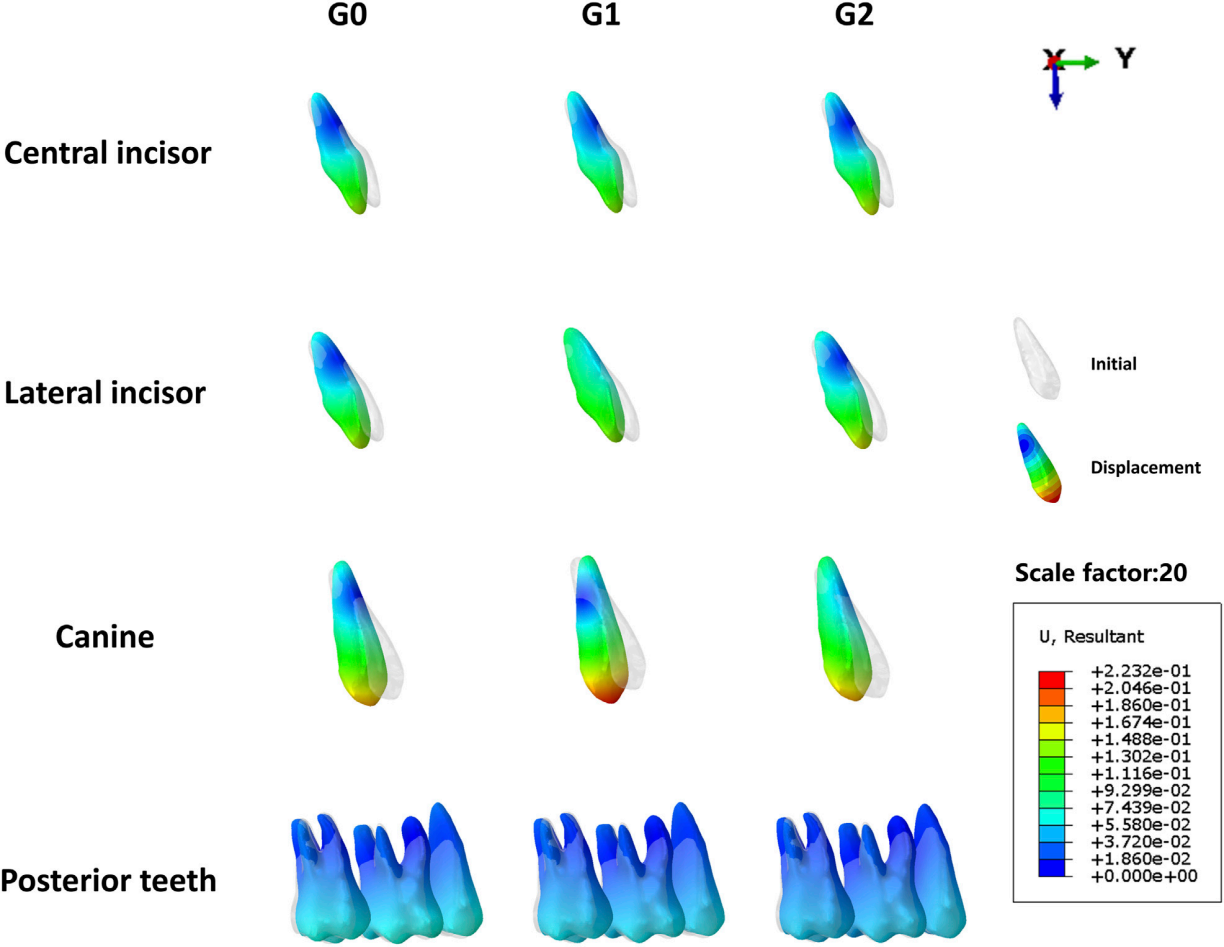
Displacement tendencies at the end of the iteration of individual teeth in each group. G0: control group; G1: incisor intrusion group; G2: anterior teeth intrusion group.

**FIGURE 5:**
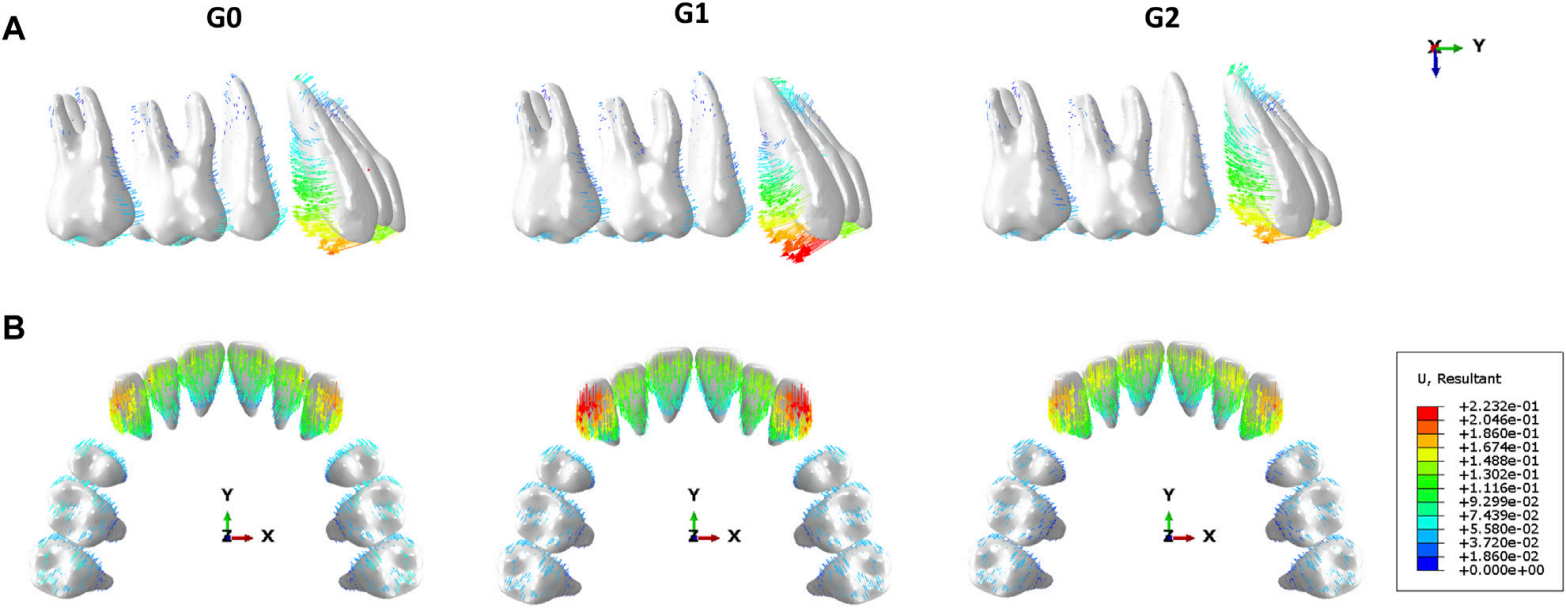
Vector diagram showing tooth displacement direction and distance at the end of the iteration in each group. **(A)** Lateral view. **(B)** Occlusal view. G0: control group; G1: incisor intrusion group; G2: anterior teeth intrusion group.

**FIGURE 6:**
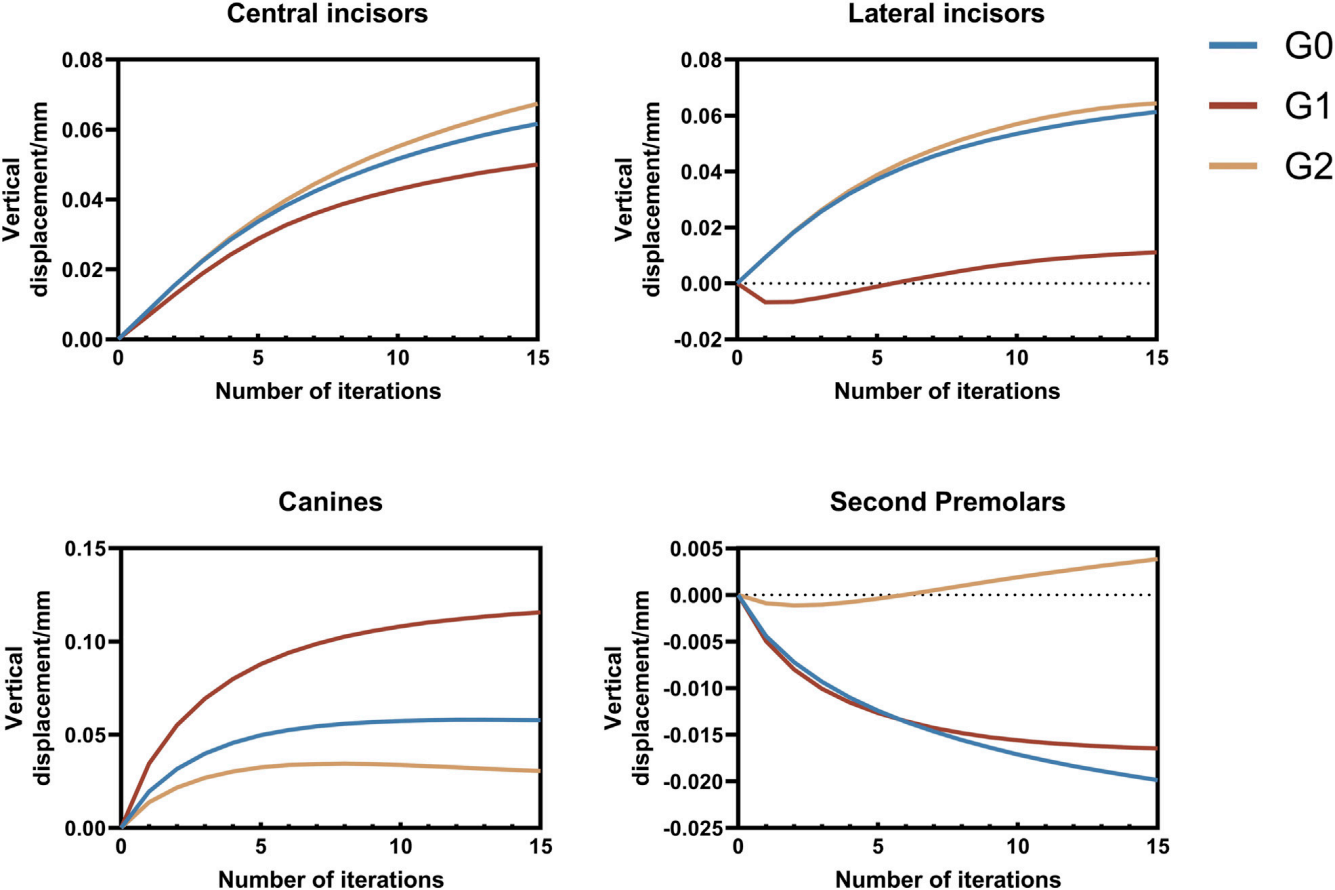
Vertical displacement of individual teeth varying with each iteration. Positive values indicate extrusion, while negative values indicate intrusion. G0: control group; G1: incisor intrusion group; G2: anterior teeth intrusion group.

**FIGURE 7:**
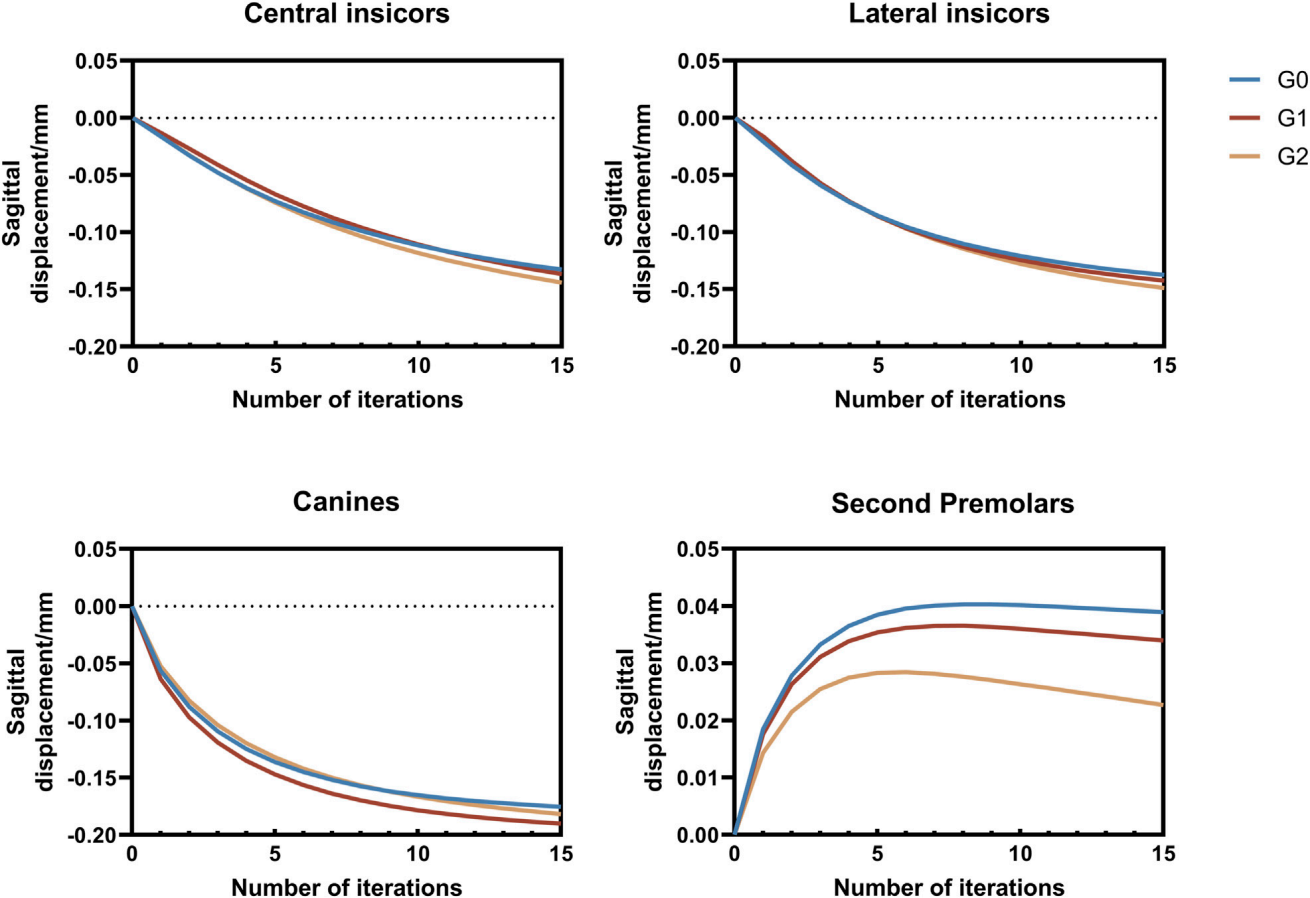
Sagittal displacement of individual teeth varying with each iteration. Positive values indicate lingual or distal displacement, whereas negative values indicate labial or distal displacement. G0: control group; G1: incisor intrusion group; G2: anterior teeth intrusion group.

In this study, the maxillary dentition was symmetrical; therefore, the right maxillary dentition was used for the analysis. The displacement tendencies of the teeth and dentition in the three groups are shown in Figures 4, 5. The amount of vertical and sagittal displacement of individual teeth varied with each iteration, as shown in Figures 6, 7.

### 3.1 Analysis of tooth vertical displacement with iterations

Extrusion of the central incisors was observed in all three groups. The extrusion of the central incisors gradually increased with each iteration, whereas the increasing tendency was gradually moderated. The extrusion of central incisors in three groups at the end of iteration followed a descending order: G2 (0.067 mm)>G0 (0.061 mm) >G1 (0.050 mm).

The lateral incisors of all three groups exhibited extrusion movement at the end of iteration, with the G1 showing a significantly smaller extrusion tendency than that in the other two groups. Additionally, it is worth noting that the lateral incisors of G1 initially showed intrusion before exhibiting an extrusion tendency after six iterations. The extrusion of lateral incisors in three groups at the end of iteration followed a descending order: G2 (0.064 mm)>G0 (0.061 mm) >G1 (0.011 mm).

The extrusion of canines in three groups at the end of iteration followed a descending order: G1 (0.12 mm)>G0 (0.058 mm)>G2 (0.031 mm), with the increasing tendency gradually diminishing as the number of iterations increased. Notably, the G2 exhibited a tendency toward intrusion.

Initial observations revealed mesial inclination and intrusion of the second premolars in all three groups. However, after more than seven iterations, extrusion movement was observed, specifically in the second premolar of the G2. At the end of the iteration, the intrusion of the second premolar in G0 was 0.020 mm, greater than that in G1, which was 0.016 mm, whereas the extrusion in G2 amounted to 0.0038 mm. (Figure 6).

### 3.2 Relative vertical displacement of anterior teeth at the end of iteration

**FIGURE 8:**
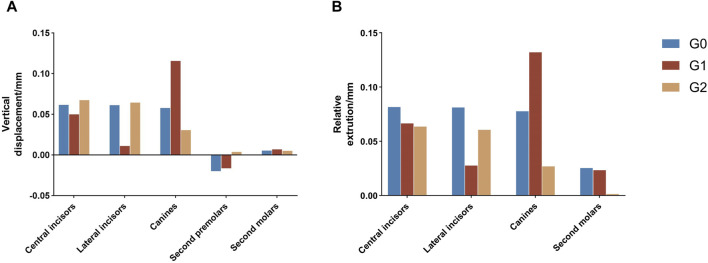
**(A)** Vertical displacement of individual teeth at the end of iteration. **(B)** Vertical displacement of individual teeth relative to the second premolar. Positive values indicate tooth extrusion. Negative values indicate tooth intrusion. G0: control group; G1: incisor intrusion group; G2: anterior teeth intrusion group.

The incisors in the three groups exhibited a relative extrusion pattern, with G0 (0.082 mm) showing the highest degree of extrusion, followed by G1 (0.066 mm) and G2 (0.064 mm). The lateral incisors exhibited an extrusion trend in the three groups, with G0 (0.081 mm) demonstrating the greatest extrusion amount, followed by G2 (0.061 mm) and G1 (0.028 mm). The relative extrusion of the canines in the three groups followed the order G1 (0.13 mm) >G0 (0.078 mm) >G2 (0.027 mm) (Figure 8).

### 3.3 The rotational tendency of the occlusal planes at the end of iteration

**FIGURE 9:**
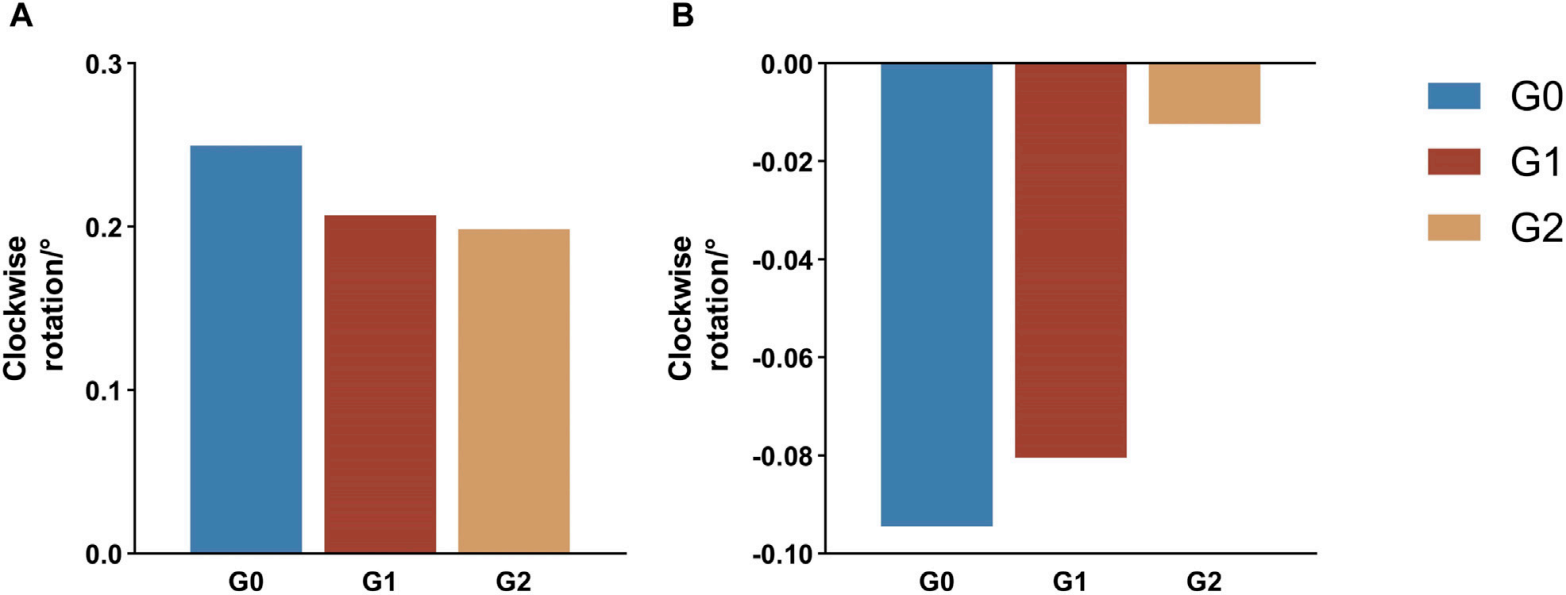
Rotation tendency of the occlusal planes at the end of iteration. A positive value indicates a tendency for clockwise rotation, whereas a negative value indicates a trend toward a counterclockwise rotation. **(A)** Anterior occlusal plane **(B)** Posterior occlusal plane G0: control group; G1: incisor intrusion group; G2: anterior teeth intrusion group.

In this study, the AOP in all three groups exhibited clockwise rotation, with the rotation angle decreasing in the following order: G0 (0.25°) > G1 (0.21°) > G2 (0.20°). The POP in all three groups exhibited a counterclockwise rotation, with G0 (0.094°) displaying the greatest rotation angle, whereas G2 (0.012°) demonstrated a significantly smaller angle than the other two groups (Figure 9).

## 4 Discussion

### 4.1 Loading methods of CAs in FEA

The biomechanics of CA remain a research hotspot in the field of orthodontics. FEA is widely used as a reliable method in this field, with the accuracy of the results obtained being influenced by factors such as the geometric model quality, material parameter accuracy, and loading mode. CAs are thermoformed, removable orthodontic appliances made from polymetric sheets, which exhibit variable thickness due to their fabrication process. Zhu et al. determined that the mean thickness of CA is approximately 0.5 mm and, using finite element analysis, demonstrated that a model simplified to this average thickness achieves reliable results (Zhu et al., 2023). Consequently, this study used a CA model with a standardized thickness of 0.5 mm.

Previous studies have shown variations in the loading methods for CAs, including stress redistribution (Jiang et al., 2020), interference fit (Fan et al., 2022), thermal contraction (Liu et al., 2023a), displacement load (Cardona et al., 2015) and birth-death elements (Hong et al., 2021), all of which are recognized for their effectiveness. Based on a previous study (Jiang et al., 2020), the loading method was further optimized by proposing a new approach based on the initial state field. Compared with previous studies, the method presented in this study more accurately emulates the application of CAs onto the dentition and demonstrates superior convergence and accuracy. In this study, this method was demonstrated to be applicable to the iterative simulation of OTM in CAT. Importantly, it was shown that the forces exerted on the teeth by the CAs can vary with tooth displacement.

### 4.2 Tendency of displacement shifted with each iteration

**FIGURE 10:**
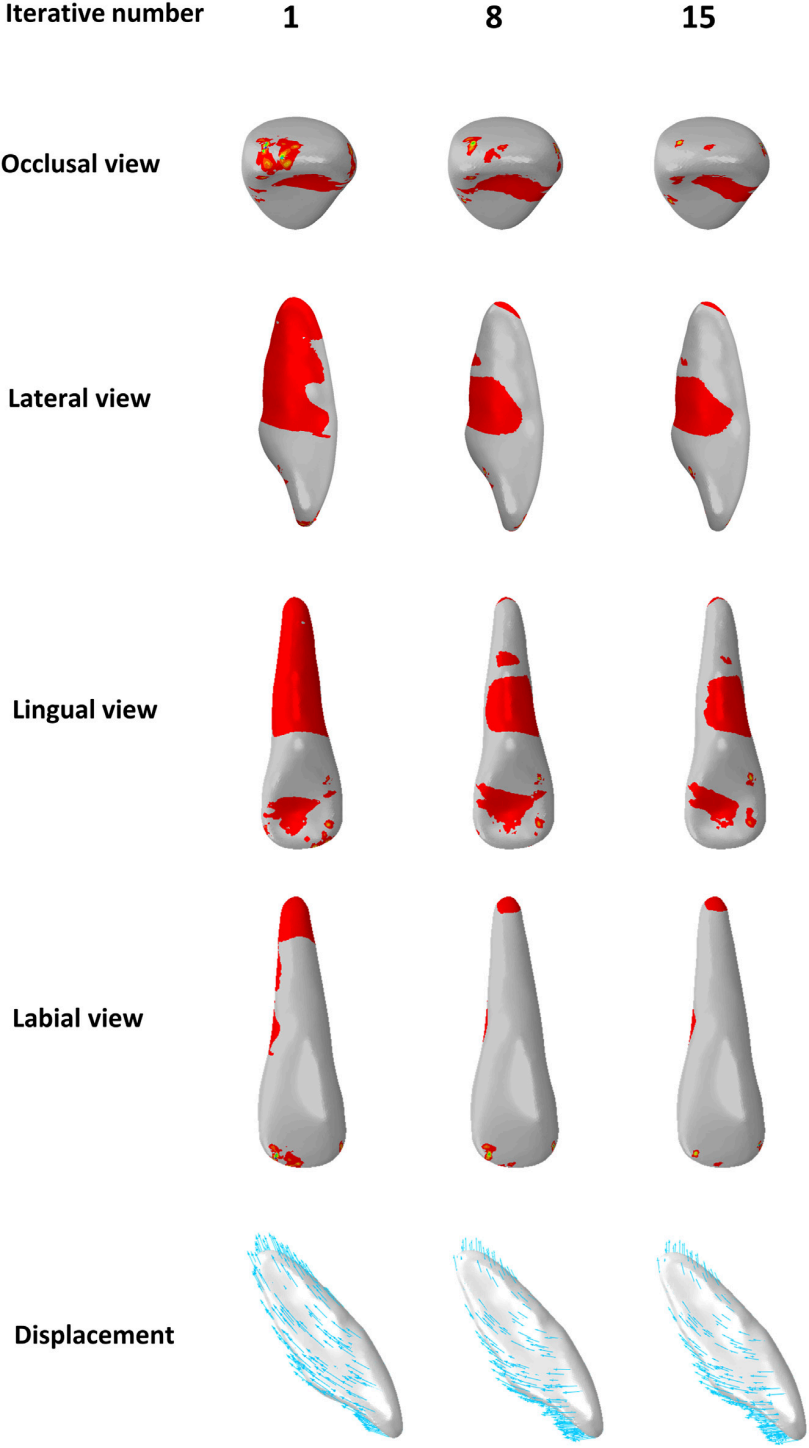
Distribution of pressure areas and displacement tendencies of the lateral incisors in the incisors intrusion group (G1) with different iterative numbers.

**FIGURE 11:**
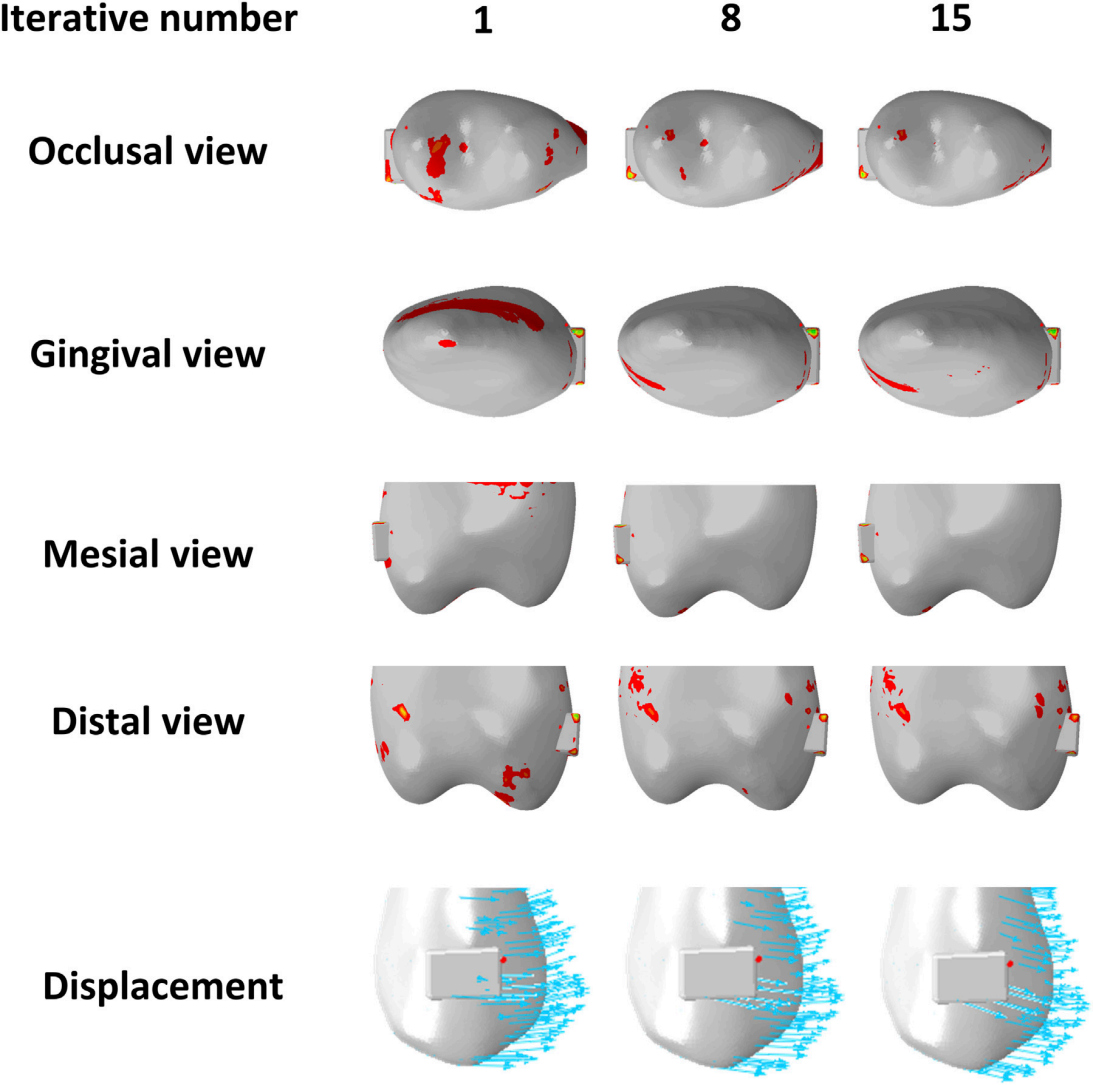
Distribution of pressure areas and displacement tendencies of the second premolar in the anterior teeth intrusion group (G2) with different iterative numbers.

Most previous studies applied a single-step static analysis to simulate initial tooth displacement when using a CA. An *in vivo* study based on fixed appliances also found that initial tooth displacement is a predictor of long-term OTM (Soenen et al., 1999). However, the biomechanics of CAs are complex, and the biomechanical effects of CAT on dentition occur over a prolonged period of time. The position of the teeth and the shape of the CA change with the remodeling of the alveolar bone (Lyu et al., 2023), which impacts the displacement tendencies of the teeth. However, it is difficult to simulate alveolar bone remodeling based on the law of biological bone remodeling (Kawamura et al., 2023). In the present study, we used the method proposed by Hamanaka et al. to simulate OTM (Hamanaka et al., 2017); however, as the authors highlighted, the iterative steps of this simulation method did not match the clinical treatment time. In a previous study, Liu et al. (Hu and Liu, 2018) found that the effect exerted by CA on teeth depended on the actual shape variables of the CA, and we observed a consistent phenomenon during simulated OTM in the present study. In this study, the lateral incisor in the G1 group was initially intruded, but became extruded after several iterations. The observed phenomenon may be attributed to the gradual increase in the amount of extrusion of both the central incisors and canines with each iteration, resulting in reduced pressure on the lateral incisor ridge and inadequate expression of the intrusion of the CA in the lateral incisor area (Figure 10). Similarly, the second premolars in G2 group exhibited comparable changes to those observed in the lateral incisors of group G1 (Figure 11), possibly due to the increased amount of intrusion relative to the extrusion of the canines, leading to a shift in the vertical displacement tendency for the second premolar. The biomechanical effects of CA on teeth are dynamic processes that depend on the shape variable at that time and are closely related to the movement of adjacent teeth. In summary, displacement tendency of teeth is subject to change over the course of wear time, necessitating a reassessment of the accuracy of utilizing initial displacement for predicting OTM in CAT.

### 4.3 The impact of overtreatment on tooth displacement

In this study, we designed two different overtreatment patterns during the bodily retraction of anterior teeth, i.e., the bodily intrusion of the incisors (G1) and anterior teeth (G2). By following the recommended step distance of CAT, we were able to control the intrusion amount so that the total displacement amounts of individual teeth did not exceed 0.25 mm, and the influence of different intrusion patterns on tooth displacement tendency during anterior tooth retraction was analyzed by the cumulative displacement at the last iteration. When the incisors were intruded, controlled vertical extrusion was observed in the lateral incisors, which manifested as controllable extrusion and inclined tendency. Compared to the control group, a reduction in the extrusion tendency was observed in the central incisors, characterized by an inclined motion of extrusion. Jiang et al. (Jiang et al., 2020) realized the ideal vertical control of the incisors by intrusion of the incisors during bodily retraction; however, in this study, vertical control of the central incisors was not achieved despite applying equal amounts of intrusion to the incisors, possibly due to the difference in retraction patterns. The mismatch at the canines diminished the expression of incisor intrusion. Distal inclination and extrusion were more significant in the canines compared to the control group. When the anterior teeth were intruded during bodily retraction, the inclination tendency of the canines and second premolars decreased, while the extrusion and sagittal displacement tendencies of incisors increased (Figures 6, 7). This phenomenon may be attributed to the reduced extrusion of the canines, resulting in reduced CA escape in the incisor area and an enhanced efficacy. Although this intrusion pattern alleviates the roller-coaster effect in the canines and second premolars, achieving bodily movement within the recommended step range remains unattainable. Overall, our findings regarding maxillary dentition are consistent with those of Zhu et al. (Zhu et al., 2021).

### 4.4 The rotational tendency of the occlusal planes

The occlusal plane is usually defined as a straight line from the tip of the central incisor to the occlusal surface of the first molar. However, it is not a merely straight line (Zhu et al., 2022). To describe this curve more precisely, Fushima (Fushima et al., 1996) divided the maxillary occlusal plane into the AOP and POP. The posterior maxillary occlusal plane was found to be related to the position and shape of the mandible, and the POP generally became steeper when the mandible was retracted. In this study, we used the rotation angles of AOP and POP to evaluate the effects of anterior teeth overtreatment patterns on the occlusal plane. In G1, the incisors were intruded during retraction of the anterior teeth, resulting in minimal extrusion of the incisors and counterclockwise rotation of the POP. In the G2 group, when the anterior teeth were intruded, the rotation angle of the POP was close to 0, and the vertical movement of the canines and second premolars was effectively controlled, significantly reducing the inclination tendency of the middle dentition. Fushima noted that it was essential to improve the Class II malocclusion by correcting the steep POP to control the vertical height of the molars. Ye et al. (Zhao et al., 2013) reported that the steep occlusal plane of patients with Class II malocclusion was mainly related to over-eruption of the incisors, but not to the height of the molar area. Zhu’s study (Zhu et al., 2022) showed that in orthodontic cases with four premolars extracted, AOP-SN and AOP-POP had a greater increase in inclination than in non-extraction cases, which could lead to rotation of the maxilla-mandible complex, which in turn could exert an impact on aesthetics and function. Therefore, the effect of changes in the occlusal plane on facial profile should be considered during orthodontic treatment. Specifically, the adoption of an appropriate overtreatment pattern based on the clinical condition of the patient is essential, with the aim of optimizing the biomechanical expression of CAs and enhancing CAT effectiveness on tooth displacement.

It should be noted that, in this study, within the recommended step range, overtreatment by solely increasing the amount of anterior teeth intrusion did not fully counteract the roller-coaster effect during bodily retraction of the anterior teeth nor the reverse rotation of AOP and POP. Therefore, additional auxiliary devices may be necessary to enhance vertical control in treatment, based on individual patient circumstances. As the most commonly used device for maxillary anterior teeth retraction, Class II elastic traction could lead to disengagement in the anterior region, resulting in greater extrusion and labial tipping of the anterior teeth, as well as clockwise rotation of the occlusal plane (Kwak et al., 2023). Moreover, the direction of the traction force could lead to a vertical effect that potentially diminishes the efficacy of intrusion overtreatment in the retraction of anterior teeth. Secondly, the mini-screw, a common auxiliary anchorage device, can produce different biomechanical effects when paired with elastic traction at different sites. Previous studies have shown that an anterior mini-screw with intrusive elastics was able to effectively achieve incisor intrusion and palatal root torquing, with linguoincisal elastics being more advantageous than labial elastics (Liu et al., 2021). When the mini-screw is located at the molar area, indirect strong anchorage can deepen the longitudinal occlusal curve, which is more suitable for patients with an open bite or shallow overbite of the anterior teeth. However, direct anchorage with elastic force is conducive to flattening the longitudinal occlusal curve, which is suitable for patients with a normal or deep overbite of the anterior teeth (Zhu et al., 2023). Lastly, the torque ridge should also be considered. Previous study has shown that the torque ridge can effectively reduce the palatal tipping tendency of the anterior teeth (Cheng et al., 2022a).

### 4.5 Limitations

As with previous iterative FEA studies, the primary limitation of this study is that the number of iterations cannot exactly correspond to CA wear time. Furthermore, CA materials in this study were consistently identical to their initial condition, and factors such as stress relaxation and material aging caused by saliva and occlusal forces were not considered. In addition, this study only examined the biomechanical effects of overcorrection in different anterior teeth intrusion patterns and did not consider the roles of anterior tooth torque and auxiliary devices. These aspects warrant further investigation. Finally, individual compliance and biological response to orthodontic forces may also impact treatment outcomes. Therefore, clinical studies are necessary to further validate the findings of this study.

## 5 Conclusion

Despite the limitations, we can draw the following conclusion:

1. In CAT, tooth displacement tendency may change with increased wear time.
2. During anterior teeth retraction, the incisor intrusion pattern can have an effective vertical control on the lateral incisors but has minimal effect on the central incisors. The anterior teeth intrusion pattern can alleviate the inclination of the canines and second premolars, resulting in partial relief of the roller-coaster effect.

## Data Availability

The original contributions presented in the study are included in the article/Supplementary material, further inquiries can be directed to the corresponding authors.

## Abbreviations

CAT, clear aligner treatment; CA, clear aligner; FEA, finite element analysis; OTM, orthodontic tooth movement; CBCT, cone beam computed tomography; PDL, periodontal ligament; AOP, anterior occlusal plane; POP, posterior occlusal plane.
